# Alcoholic Odor of the Fluid in the Ventricles of the Brain

**Published:** 1845-01

**Authors:** 


*Alcoholic Odor of the Fluid in the Ventricles of the Brain.* Dr. Bradley, of Illinois, was summoned before a coroner's inquest, held on the body of Samuel Page, as a medical witness, The deceased was found about two miles from the village, in his wagon, with his feet hanging over the foreboard, his body resting upon a bag of grain, and his head upon the bottom of the wagon. He was totally insensible, and his breathing stertorous and difficult. He was taken to a neighboring dwelling, where he died in about ten minutes. He had been drinking very freely for several days previous, but was not an habitual drunkard. He had left a grocery store a short time previous to being found, partially intoxicated, without mittens or any other "extra over-clothes," though the weather was somewhat below the freezing point.

On dissection, six hours after death, dark fluid blood poured forth from the sinuses of the brain, to the amount of eight or ten ounces. The brain exhibited excessive vascular turgescence; in the corpora striata, a small amount of sanguineous extravasation was detected, and in the lateral ventricles some serous effusion. Verdict, "death from apoplexy caused by intemperance."

*The effused fluid found in the ventricles yielded strongly the alcoholic odor.* This was so apparent, that it was readily recognised by every member of the jury.

*Ill. Med. and Surg. Jour.*

